# Effect of Staged Preconditioning on Biochemical Markers in the Patients Undergoing Coronary Artery Bypass Grafting

**DOI:** 10.5402/2012/204624

**Published:** 2012-08-02

**Authors:** Alireza Mohammadzadeh, Naser Jafari, Behzad Babapoursaatlou, Hossein Doustkami, Adallat Hosseinian, Mohammad Hasanpour

**Affiliations:** ^1^Department of Cardiothoracic, Imam Khomeini Hospital, Ardabil 56197, Iran; ^2^Department of Biochemistry, School of Medicine, Ardabil University of Medical Sciences, Ardabil 56197, Iran

## Abstract

The present study has investigated the effectiveness of staged-preconditioning, in both remote and target organs. After IP the myocardial release of the biochemical markers including, creatine phosphokinase (CPK), cardiac creatine kinase (CK-MB), cardiac troponin T (cTnT) and lactate dehydrogenase (LDH) were evaluated in patients who underwent CABG, with and without staged-preconditioning. Sixty-one patients entered the study; there were 32 patients in the staged-preconditioning group and 29 patients in the control group. All patients underwent on-pump CABG using cardiopulmonary bypass (CPB) techniques. In the staged-preconditioning group, patients underwent two stages of IP on remote (upper limb) and target organs. Each stage of preconditioning was carried out by 3 cycles of ischemia and then reperfusion. Serum levels of biochemical markers were immediately measured postoperatively at 24, 48 and 72 h. Serum CK-MB, CPK and LDH levels were significantly lower in the staged-preconditioning group than in the control group. The CK-MB release in the staged-preconditioning patients reduced by 51% in comparison with controls over 72 h after CABG. These results suggest that myocardial injury was attenuated by the effect of three rounds of both remote and target organ IP.

## 1. Introduction

New treatment strategies are required to reduce myocardial injury and improve clinical outcomes in patients with coronary heart disease (CHD), the leading cause of death worldwide. 

The protective effects of ischemic preconditioning (IP) on human heart after cardiac surgeries are well defined [1]. Initially, the positive effect of short periods of ischemia to limit infarct size was referred to IP [2]. It was then established to have beneficial effects on ischemia and reperfusion induced arrhythmia and on myocardial stunning [3, 4]. Furthermore, it has been suggested that IP reduces postoperative inotropic requirements and has a preventive role in postoperative myocardial dysfunction [5, 6].

Ischemic preconditioning can be mechanical or pharmacological. Direct mechanical preconditioning in which the major organ is exposed to brief ischemia prior to prolonged ischemia has the benefit of reducing ischemia-reperfusion injury (IRJ). Remote preconditioning is a brief ischemia of one organ which has been shown to confer protection on distant organs without direct stress to the target organ [7].

Remote ischemic preconditioning (RIPC) can limit myocardial infarction size that has been successfully applied to the protection of other noncardiac organs against ischemia-reperfusion injury [8]. Both types of precondition have their own advantages. In this regard, we have investigated the effectiveness of staged-preconditioning including both remote and target organ ischemic preconditioning on patients undergoing on-pump coronary artery bypass grafting (CABG) using cardiopulmonary bypass (CPB) method. The present study evaluated the myocardial release of the cardiac biochemical markers including, creatine phosphokinase (CPK), cardiac creatine kinase (CK-MB), cardiac troponinT (cTnT), and lactate dehydrogenase (LDH) in patients undergoing CABG, with and without staged-preconditioning. These biochemical markers are widely used to detect postoperative myocardial damage during CABG surgery [9].

## 2. Patients and Methods

This randomized controlled trial was carried out at Imam khomeini Hospital, Ardabil, Iran. An ethics committee of Human research of Ardabil University of Medical Sciences approved the study. Measurement of biochemical markers after cardiovascular surgeries is routine and all patients gave an informed consent before enrolment.

*Patients*. Sixty-one patients (male/female: 33/28, mean age: 58 ± 10 years), who underwent first-time elective CABG entered the study; there were 32 patients in the staged preconditioning group and 29 patients in the control group. Patients were excluded if they had concomitant disease of heart valves, pulmonary hypertension, myocardial infarction (MI), and history of unstable angina within the past two months and preoperative inotropic support or any kind of mechanical assist device.

*Surgical Techniques*. All patients underwent on-pump coronary artery bypass grafting (CABG) using standard cardiopulmonary bypass (CPB) techniques with antegrade and retrograde bloody cardioplegia. The anesthetic and CABG techniques were the same in all cases. In the staged-preconditioning group, patients underwent two stages of ischemic preconditioning at remote (upper limb) and target organs. After induction of anesthesia, remote ischemic preconditioning (RIPC) was carried out by 3 cycles of 5 min left upper arm ischemia by inflation of a blood pressure cuff to 200 mmHg and then reperfusion. First ischemia before preparation of the patients, second ischemia after sternotomy, and third ischemia was achieved just performing left internal mammary artery (LIMA). Second stage of preconditioning was carried out on the target organ in 3 cycles too. During each phase the pressure was established between 30 to 40 mmHg for 1.5 min. First ischemia after cannulation and just before CPB, second phase after CPB and before cardioplegia perfusion, and third phase of ischemia was induced just performing declamping.

*Blood Sampling and Biochemical Markers*. Venous blood samples were drown from each patient in two groups postoperatively at 24, 48, and 72 h and analyzed separately for CPK, its cardiac isoenzyme (CK-MB), cTnT, and LDH. All samples were immediately centrifuged for 10min at 3000 g(rcf), and serum enzyme activities were measured at 37°C. Serum CPK, CK-MB, and LDH determinations were done by means of test kits (Parsazmoon, Iran) using an autoanalyzer system (BT3000, Italy). Determination of serum cTnT was performed by means of test kit (Elecsys2010, Switzerland) using electrochemiluminescence immunoassay (ECLIA).

The reference interval for CPK and LDH is 24–170 u/l and 150–500 u/l, respectively. The upper limits of the reference interval for the CK-MB and cTnT assays are 24 u/l and 0.01 ng/l, respectively.

*Statistical Analysis*. Statistical analysis was performed using a graph and data analysis software package (SigmaPlot 11.0, Systat Software, Inc.). Data are presented as mean ± SD, except in figures where error bars represent SEM. ANOVA tests followed by the pairwise Student-Newman-Keuls test for multiple comparisons were performed to check for differences. *P* value less than 0.05 was considered significant.

## 3. Results

There was no recorded case of mortality or morbidity in these two groups of patients. There were no significant differences between the two groups with regard to preoperative parameters such as sex, age, and body surface area. Cumulative cardiac biochemical marker release was calculated as the mean net release at all 3 measuring time points after operation.

**Figure 1:**
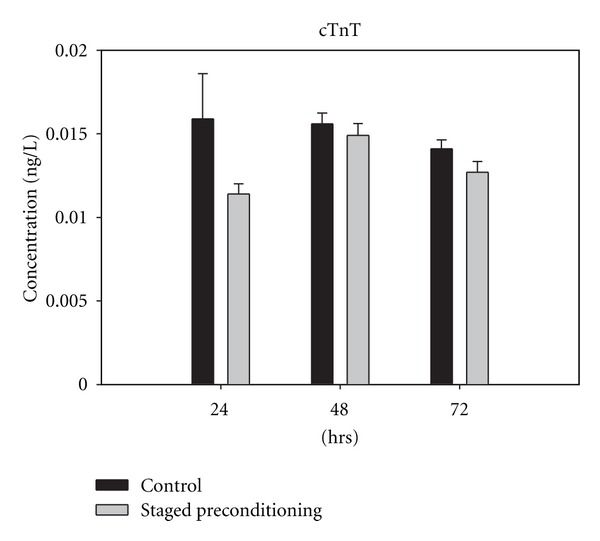
Cardiac troponint (cTnT) concentration in control and staged preconditioning groups, 24 h, 48 h, and 72 h after CABG. The differences in the median values among the control and case groups are greater than would be expected by chance; there is a statistically significant difference, all pairwise multiple comparisons showed *P* value less than 0.05.

Serum concentrations of these biochemical markers increased postoperatively and reached a peak level at 48 h after operation in both groups, except for cTnT in the control group that had a peak level at 24 hrs after surgery (Figure 1).

**Figure 2:**
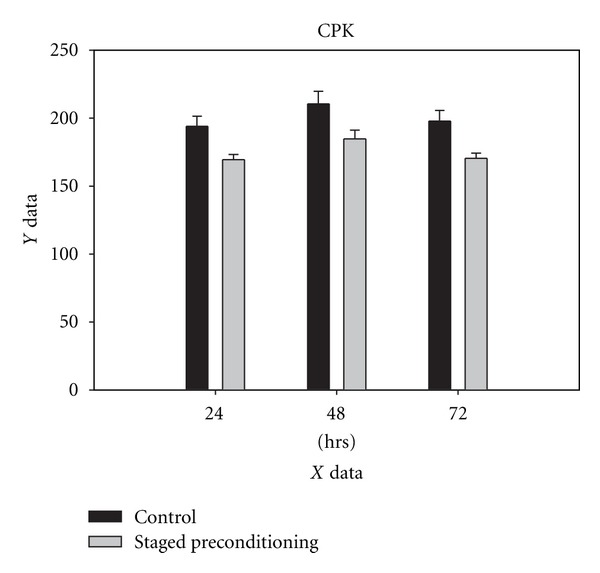
Creatine phosphokinase (CPK) concentration in control and staged preconditioning groups, 24 h, 48 h, and 72 h after CABG, all pairwise multiple comparisons showed *P* value less than 0.05.

**Figure 3:**
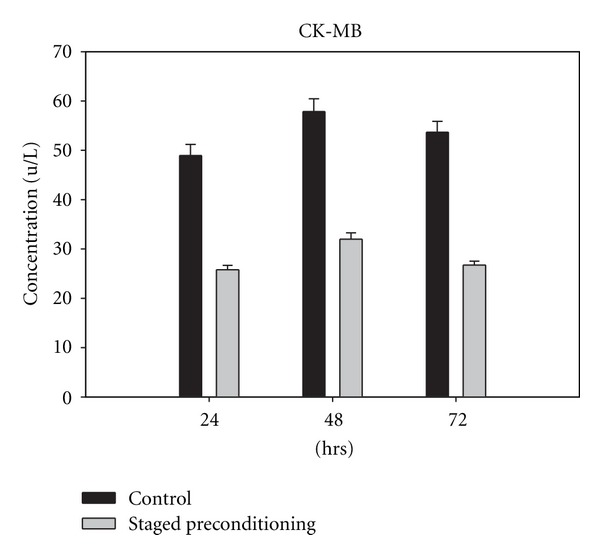
Cardiac creatine kinase (CK-MB) concentration in control and staged preconditioning groups, 24 h, 48 h, and 72 h after CABG, all pairwise multiple comparisons showed *P* value less than 0.05.

**Figure 4:**
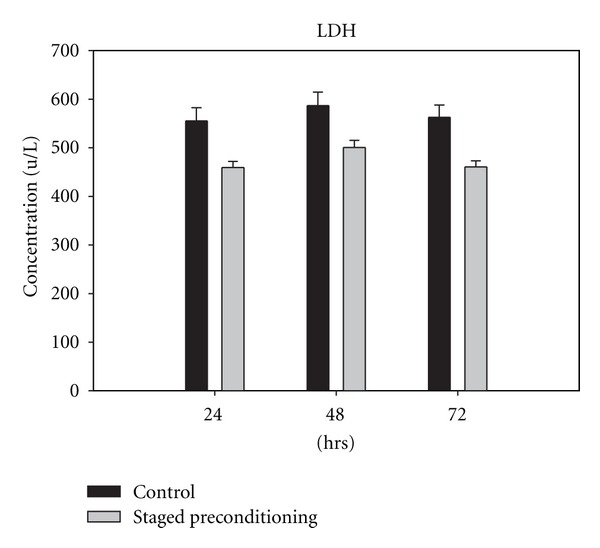
Lactate dehydrogenase (LDH) concentration in control and staged preconditioning groups, 24 h, 48 h, and 72 h after CABG, all pairwise multiple comparisons showed *P* value less than 0.05.

The concentration of CPK, CK-MB, and LDH rose significantly in the control group within 24, 48, and 72 h compared with the baseline values obtained from staged-precondition group at the same time points (Figures 2, 3, and 4). Levels of CK-MB postoperatively were markedly greater in the control patients as compared with the staged preconditioning group (*P* = 0.001 in three time points) indicating greater myocardial injury in controls (Figure 3). The CK-MB release in the staged preconditioning patients reduced by 51% in comparison with controls over 72 h after CABG.

In contrast to CPK, CK-MB, and LDH, the difference between control and staged preconditioning groups release values for cTnT at 48 and 72 h after operation was not significant. The rate of increase for cTnT at 24 h was significantly higher (*P* = 0.04) than that at 48 and 72 hrs in the control group compared with the staged preconditioning group (Figure 1).

## 4. Discussion

It has been shown that the direct application of an IP stimulus protects patients against irreversible myocardial injury [10]. The use of IP with repeated cycles of ischemic stimuli has been proposed to ensure the protective effects [11]. It became evident that RIPC provides an easy and safe method of increasing myocardial surgeries [12]. RIPC with transient upper limb ischemia and reperfusion were shown to reduce myocardial injury in patients undergoing CABG with intermittent coldblood cardioplegia for myocardial protection [12, 13]. Each kind of preconditioning by inducing brief ischemia before operation increases the body's resistance and has a protective effect after surgery [14, 15]. In this regard, preliminary studies have not been investigated the concomitant application and effectiveness of remote and target organ IP in patients undergoing CABG.

In this study we have evaluated the effect of staged preconditioning on the release of cardiac biochemical markers in patients undergoing CABG. The novel and unique approach of this study was to measure lower levels of these biochemical markers in patients underwent staged preconditioning. In this study serum CK-MB, CPK, and LDH levels were significantly lower in staged preconditioning group than in the control group. We have demonstrated that leakage of CK-MB, CPK, LDH, and cTnT were inhibited to varying degrees in the staged preconditioning group at several postoperative time points compared to the control group. The high levels of CK-MB in control patients compared with staged preconditioning patients were remarkable. In our study, 51% reduction of CK-MB release in staged preconditioning group was achieved over 72 h after CABG. These results suggest that myocardial injury was attenuated by the effect of three rounds of both remote and target organs IP. Consequently, the higher levels of biochemical markers in the control group can be interpreted as reflecting a greater degree of myocardial injury. The results of this study indicate that better protection after CABG can be explained by the overlap of the two widows of protection derived from the two types of IP treatments. These findings underscore the effectiveness and protective effects of the application of IP methods in both remote and target organs. Whether or not such protection by staged preconditioning has beneficial impact on patient's short- and long-term outcome will have to be elucidated in the future large-scale studies.

## References

[1] Teoh LKK, Grant R, Hulf JA, Pugsley WB, Yellon DM (2002). A comparison between ischemic preconditioning, intermittent cross-clamp fibrillation and cold crystalloid cardioplegia for myocardial protection during coronary artery bypass graft surgery. *Cardiovascular Surgery*.

[2] Murry CE, Jennings RB, Reimer KA (1986). Preconditioning with ischemia: a delay of lethal cell injury in ischemic myocardium. *Circulation*.

[3] Shiki K, Hearse DJ (1987). Preconditioning of ischemic myocardium: reperfusion-induced arrhythmias. *American Journal of Physiology*.

[4] Cohen MV, Liu GS, Downey JM (1991). Preconditioning causes improved wall motion as well as smaller infarcts after transient coronary occlusion in rabbits. *Circulation*.

[5] Wu ZK, Tarkka MR, Pehkonen E, Kaukinen L, Honkonen EL, Kaukinen S (2000). Ischaemic preconditioning has a beneficial effect on left ventricular haemodynamic function after a coronary artery biopass grafting operation. *Scandinavian Cardiovascular Journal*.

[6] Wu ZK, Vikman S, Laurikka J (2005). Nonlinear heart rate variability in CABG patients and the preconditioning effect. *European Journal of Cardio-Thoracic Surgery*.

[7] Tapuria N, Kumar Y, Habib MM, Amara MA, Seifalian AM, Davidson BR (2008). Remote ischemic preconditioning: a novel protective method from ischemia reperfusion injury—a review. *Journal of Surgical Research*.

[8] Hausenloy DJ, Yellon DM (2008). Remote ischaemic preconditioning: underlying mechanisms and clinical application. *Cardiovascular Research*.

[9] Saikia MK, Rupert E, Muralidhar K, Shetty DP (2000). Biochemical markers of myocardial injury during coronary artery bypass grafting with and without cardiopulmonary bypass. *Annals of Cardiac Anaesthesia*.

[10] Schott RJ, Rohmann S, Braun ER, Schaper W (1990). Ischemic preconditioning reduces infarct size in swine myocardium. *Circulation Research*.

[11] Downey JM, Cohen MV (1997). Signal transduction in ischemic preconditioning. *Advances in Experimental Medicine and Biology*.

[12] Thielmann M, Kottenberg E, Boengler K (2010). Remote ischemic preconditioning reduces myocardial injury after coronary artery bypass surgery with crystalloid cardioplegic arrest. *Basic Research in Cardiology*.

[13] Maslov LN, Lishmanov IB (2009). Cardioprotective effect of remote ischemic preconditioning. *Angiologiia i Sosudistaia khirurgiia*.

[14] Takagi H, Manabe H, Kawai N, Goto SN, Umemoto T (2008). Review and meta-analysis of randomized controlled clinical trials of remote ischemic preconditioning in cardiovascular surgery. *American Journal of Cardiology*.

[15] Eisen A, Fisman EZ, Rubenfire M (2004). Ischemic preconditioning: nearly two decades of research. A comprehensive review. *Atherosclerosis*.

